# An Encapsulated *Yersinia pseudotuberculosis* Is a Highly Efficient Vaccine against Pneumonic Plague

**DOI:** 10.1371/journal.pntd.0001528

**Published:** 2012-02-14

**Authors:** Anne Derbise, Alba Cerdà Marín, Patrick Ave, Thierry Blisnick, Michel Huerre, Elisabeth Carniel, Christian E. Demeure

**Affiliations:** 1 Unité de Recherche Yersinia, Institut Pasteur, Paris, France; 2 Unité Histotechnologie et Pathologie, Institut Pasteur, Paris, France; University of Tennessee, United States of America

## Abstract

**Background:**

Plague is still a public health problem in the world and is re-emerging, but no efficient vaccine is available. We previously reported that oral inoculation of a live attenuated *Yersinia pseudotuberculosis*, the recent ancestor of *Yersinia pestis*, provided protection against bubonic plague. However, the strain poorly protected against pneumonic plague, the most deadly and contagious form of the disease, and was not genetically defined.

**Methodology and Principal Findings:**

The sequenced *Y. pseudotuberculosis* IP32953 has been irreversibly attenuated by deletion of genes encoding three essential virulence factors. An encapsulated *Y. pseudotuberculosis* was generated by cloning the *Y. pestis* F1-encoding *caf* operon and expressing it in the attenuated strain. The new V674pF1 strain produced the F1 capsule *in vitro* and *in vivo*. Oral inoculation of V674pF1 allowed the colonization of the gut without lesions to Peyer's patches and the spleen. Vaccination induced both humoral and cellular components of immunity, at the systemic (IgG and Th1 cells) and the mucosal levels (IgA and Th17 cells). A single oral dose conferred 100% protection against a lethal pneumonic plague challenge (33×LD_50_ of the fully virulent *Y. pestis* CO92 strain) and 94% against a high challenge dose (3,300×LD_50_). Both F1 and other *Yersinia* antigens were recognized and V674pF1 efficiently protected against a F1-negative *Y. pestis*.

**Conclusions and Significance:**

The encapsulated *Y. pseudotuberculosis* V674pF1 is an efficient live oral vaccine against pneumonic plague, and could be developed for mass vaccination in tropical endemic areas to control pneumonic plague transmission and mortality.

## Author Summary

Plague, among the most deadly infections of mankind's history, is present in Africa, Asia and America, and is currently re-emerging, recently causing cases in areas from where it had disappeared for decades. Pneumonic plague, its most deadly and contagious form, is responsible for human-to-human spreading of the infection. Vaccination would be an effective means to control the disease, but no efficient vaccine is currently available. Because live vaccines are potent inducers of protective immunity, our strategy was to use a *Yersinia pseudotuberculosis*, closely related to *Y. pestis* but genetically more stable, to make it suitable for use as live oral vaccine. We have developed a genetically defined *Y. pseudotuberculosis* strain strongly attenuated by deletion of virulence factors genes, which was also induced to produce the *Y. pestis* F1 pseudocapsule. A single oral dose was harmless and provided high- level protection against pneumonic plague. Such a candidate vaccine offers promising perspectives to control pneumonic plague mortality and transmission.

## Introduction

Plague, the dreadful infectious disease that caused three major pandemics in history, is still a public health problem. Since the 1980s, an increase of cases worldwide has been observed, leading to categorize plague as a re-emerging disease. Whereas the most active foci of human plague are located in east-central Africa and Madagascar [1], recent cases have also been recorded in areas from where it had disappeared for decades, like Algeria, Libya, Zambia and Jordan [2], [3], [4]. Moreover, because plague is principally a zoonotic disease affecting rodents, the territories where it is endemic in its animal reservoir are much more extended than the observed human plague foci.

Plague is an acute, often fatal infection whose etiologic agent, *Yersinia pestis*, is a Gram-negative bacillus with an extreme pathogenicity. The accidental transmission of the plague bacillus from rodents to humans by the bite of infected fleas is the most frequent mode of human infection. From the skin, the bacteria migrate to the draining lymph nodes, causing the bubonic form of plague, which evolves toward septicemia and death within one week if treatment is not rapidly started. When the bacteria reach the lungs, an acute pneumonia develops. Patients become highly contagious through the emission of infected aerosols that cause primary pneumonic plague. The disease is then generally fatal within 3 days or less. Human-to-human transmission of pneumonic plague favors a rapid spread of the disease in heavily populated areas, as occurred for example recently in Madagascar and in the Democratic Republic of Congo [5], [6]. Although antibiotherapy is currently the main tool to fight the disease, the residual plague mortality in endemic countries remains around 10% or more. This is mainly due to the difficulty for patients from areas far from health structures to receive the treatment on time. An additional cause of concern is the recent identification in Madagascar of two *Y. pestis* strains naturally resistant to antibiotics, one of which was resistant to eight different antibiotics, including those recommended for plague treatment and prophylaxis [7]. Because this multi-drug resistant *Y. pestis* resulted from the acquisition of a widespread self-transmissible plasmid [8], the rise of such threatening *Y. pestis* variants may be anticipated. Finally, *Y. pestis* is also classified in the list A of pathogens with potential for bioterrorist use established by the US Center for Disease Control due to its pathogenicity and human-to-human transmission [9], and the possibility that the bacteria is engineered to resist to antibiotics for evil use cannot be excluded. In front of such a public health risk, mass vaccination might be one of the only alternatives to protect exposed populations. However, no safe and efficient vaccine against plague is currently available.

The first widely used plague vaccine was the live attenuated *Y. pestis* EV76 developed in Madagascar. This vaccine, that can have severe secondary effects, is now used in only few countries such as China or the former USSR. The licensed killed whole-cell *Y. pestis* vaccine from Greer/Miles was recently discontinued because it was reactogenic in humans and conferred only short-term protection [10], requiring annual booster immunizations. Much effort has been made in the recent years to develop new candidate vaccines. The strategies followed to induce protective immunity include the attenuation of live *Y. pestis* by genetic engineering, the introduction of *Y. pestis* antigens in *Salmonella* [11] and virus vectors [12], [13], as well as the production of subunit vaccines combining the capsular F1 antigen and the V antigen (LcrV) [14], [15], [16].

We recently reported that the attenuated *Y. pseudotuberculosis* strain IP32680 can be used as a live oral vaccine against bubonic plague [17]. The rationale for choosing this approach was to combine the immunogenicity and antigenic complexity of live vaccines with the much lower virulence of *Y. pseudotuberculosis*. Indeed, *Y. pestis* is a clone recently emerged from *Y. pseudotuberculosis* [18], and the two species share more than 95% genetic identity. A reason not to use *Y. pestis* as live vaccine is its genetic instability, as revealed by the spontaneous genome reductions observed for the EV76 strain, which hampered its vaccine efficiency [19]. That risk is much lower for *Y. pseudotuberculosis* because such rearrangements in *Y. pestis* are thought to result from the high number of insertion sequences (IS) present in its genome [20], and *Y. pseudotuberculosis* has a much lower number of IS copies and so is genetically much more stable [20]. When given orally, *Y. pseudotuberculosis* IP32680 was able to colonize the gut without causing lesions and stimulated a protective immune response against bubonic plague [17]. These results demonstrate the feasibility of using a live attenuated *Y. pseudotuberculosis* strain as an oral vaccine against plague. However, IP32680 is not suitable for human use because the genetic bases of its attenuation are not known and it does not confer high-level protection against pneumonic plague.

The aim of the present study was to construct a genetically engineered *Y. pseudotuberculosis* strain irreversibly attenuated in virulence, and able to confer high-level protection against pneumonic plague.

## Materials and Methods

### Ethics statement

Animals were housed in the Institut Pasteur animal facilities accredited by the French Ministry of Agriculture to perform experiments on live mice (accreditation B 75 15-01, issued on may 22nd, 2008), in appliance of the French and European regulations on care and protection of the Laboratory Animals (EC Directive 86/609, French Law 2001-486 issued on June 6, 2001). Protocols were approved by the veterinary staff of the Institut Pasteur animal facility and were performed in compliance with the NIH Animal Welfare Insurance #A5476-01 issued on 02/07/2007.

### Bacterial strains, plasmids and culture conditions

The *Y. pseudotuberculosis* and *Y. pestis* isolates used in this study and their derivatives are listed in Table 1. Bacteria were grown at 28°C on Luria-Bertani agar plates supplemented with 0.2% hemin (LBH) for 48 h before use, and bacterial concentrations were evaluated by spectrometry at 600 nm and plating on LBH plates.

**Table 1:** Bacterial strains and plasmids used in this study.

Strain, plasmid	Relevant characteristics or sequence	Source
**CO92 derivatives**		
CO92	Wild type, biotype Orientalis	[59]
CO92p	pKOBEG-*sacB* introduced into CO92 by electroporation	[60]
CO92Δ*yopK*	CO92p deleted of *yopK*	This study
CO92Δ*psaA*	CO92p deleted of *psaA*	This study
CO92Δ*caf*	CO92p deleted of the *caf* operon	This study
**IP32953 derivatives**		
IP32953	Wild type, serotype I	[35]
IP32953p	pKOBEG-*sacB* introduced into IP32953 by electroporation	[60]
IP32953ΔHPI	IP32953p deleted of the HPI	This study
V676	IP32953p deleted of the HPI and *yopK*	This study
V674	IP32953p deleted of the HPI, *yopK* and *psaA*	This study
V674 pF1	pGEN-*caf* introduced into V674 by electroporation	[59]
***E. coli***		
TOP10	F-*mcrA* D(mrr-*hsdRMS*-*mcrBC*) f80*lacZ* DM15 DlacX74 *recA1 araD139* D(ara-leu)7697 *galU galK rpsL* (StrR) *endA1 nupG*	Invitrogen
TOP10(pGEN-*lux*)	TOP10 harboring pGEN-*lux*	[22]
TOP10(pGEN-*caf*)	TOP10 harboring pGEN-*caf*	This study
**Plasmids**		
pKOBEG-*sacB*	*repA cat araC pBAD exo bet gam sacB*, Cm^R^	[21]
pUC4K	Km^R^	Amersham
pGP704N-*km*	Suicide vector, Amp^R^, Km^R^	[61]
pGP704N-*dfr*	Suicide vector, Amp^R^, Tm^R^	[62]
pSW25	*oriT ccdB* Spec^R^	[63]
pGEN-*lux*	*hok/sok parR*/*parM bla*, *luxCDEAB*, Amp^R^	[22]
pGEN-*caf*	the *caf* operon replace the *lux* operon in pGEN-*lux*	This study

### Mutagenesis

Deletion of the *caf* operon was performed in *Y. pestis* CO92p (Table 1) using the Short Flanking Homology (SFH) procedure [21] with primers 812 and 814 designed to exchange a portion of the *caf* locus encompassing the *caf1M*, *caf1A* and *caf1* genes by a kanamycin resistance cassette (*km* from plasmid pGP704N-*km*). Deletion of the *Y. pseudotuberculosis* High Pathogenicity Island (HPI: YPTB1585 through YPTB1602), *yopK* (virulence plasmid gene PYV0040) and *psaA* (YPTB1334) sequences from IP32953p was done by allelic exchange with a kanamycin (*km* from plasmid pUC4K), spectinomycin (*spec* from plasmid pSW25) and trimethoprim (*dfr* from plasmid pGP704N-*dfr*) resistance cassette, respectively. HPI deletion was done following the Long Flanking Homology procedure [21]. *yopK* and *psaA* genes were first deleted individually in *Y. pestis* CO92p (Table 1) following the SFH procedure [21]. Second, the genomic DNA from the resulting deletants was used as template for PCR amplification of the antibiotic resistance cassettes flanked by the 500 bp upstream and downstream regions of *yopK* and *psaA* genes. The PCR products were electroporated into *Y. pseudotuberculosis* IP32953ΔHPI (Table 1), as described previously [21]. Recombinant colonies were selected for antibiotic resistance and were verified by PCR with primers located: (i) on each side of the inserted antibiotic cassette, and (ii) within each target region (Table S1). All the primer pairs used to generate PCR products for allelic exchange are listed in Table S1.

### Cloning of the *caf* operon

To clone the *caf* operon into pGEN-*lux* [22], the entire locus was amplified with primer pair 837/838, which adds *Not*I and *Apa*I sites at the extremities. The PCR product was ligated to the corresponding sites in place of the *lux* operon. Then, the ligation mix was electroporated into *E. coli* TOP10 (Invitrogen). The pGEN-*lux* plasmid was chosen because it contains the *hok*/*sok* genes coding for a toxin/antitoxin module, and the *parR*/*parM* partition system, both stabilizing the plasmid in the bacterial population [23]. The presence of the plasmid with the appropriate insert (pGEN-*caf*) was checked after plasmid extraction and digestion. The pGEN-*caf* construct was introduced by electroporation into *Y. pseudotuberculosis* V674 and recombinant strains selected. The sequence of the cloned *caf1* gene was verified by sequencing.

### Analysis of F1 production

To determine the presence of a capsule, bacteria in India ink [24] were examined by phase-contrast microscopy. To quantify the production of the F1, ELISA plates (NUNC) were coated with the anti-F1 mAb G5-18 [25], followed by a 1% BSA blocking solution in PBS. Serial dilutions of bacterial suspensions (10^9^ to 10^4^ cfu/ml) in PBS containing 0.1% BSA were laid in the wells. The plate was centrifuged for 10 min (1000 g) prior to incubation for 1 h at room temperature. After 3 washes with PBS, biotinylated G5-18 anti-F1 mAb (1 µg/ml) was incubated for 1 h, followed by 30 min with Streptavidin coupled to horseradish peroxidase (Jackson Immunoresearch), and colorimetric revelation using TMB (OptiEIA, BD-biosciences), as previously described [17].

### Animal infection and *in vivo* analyses

Mouse infections were performed in a BSL3 animal facility. Bacterial suspensions of bacteria (200 µl in saline) were given intragastrically to seven weeks old OF1 female mice (Charles River France) using a curved feeding needle. To determine the 50% lethal dose (LD_50_), mice (six per dose) were infected with 10 fold serial dilutions of bacterial suspensions and were monitored for 3 weeks. The method of Reed and Muench was used to calculate LD_50_ values [26].

*In vivo* dissemination was examined five or fifteen days after oral inoculation of bacteria. Peyer's patches, spleen and feces (two fecal pellets from the large intestine) were collected aseptically from euthanized mice and were homogenized in sterile PBS using 3 mm glass beads and an electric mill (TissueLyser®, Qiagen). The bacterial load was determined by plating serial dilutions of the homogenates.

The severity of lesions caused to tissues by *Y. pseudotuberculosis* strains was analyzed histologically. Animals were euthanized and target organs were fixed with 4% buffered formaldehyde for 48 h, embedded in paraffin, cut in 5 micrometers sections, and stained with hematoxylin–eosin. Histological sections were read blindly and lesions were quantified using a previously described scale [17] ranging from 0 to 10. For immunohistological detection of the F1 antigen produced *in vivo*, Peyer's patches collected 5 days after vaccination with the V674pF1 strain (10^10^ cfu) were fixed and embedded in low-melting point paraffin (polyEthylene Glycol distearate, Aldrich). Endogenous peroxidase activity was eliminated after deparaffinization by incubation in 0.3% hydrogen peroxide for 30 min and non-specific binding sites was blocked for 30 min, prior to incubation (1 h) with the biotinylated anti-F1 G5-18 mAb. As a specificity control, an irrelevant and isotype-matched biotinylated mAb was used. After washes in PBS, sections were incubated for 1 h with Histofine® Simple Stain MAX PO (Rabbit specific; Nichirei corp.) and peroxidase activity was detected using 3-amino ethylcarbazole (AEC) substrate (Sigma). Tissues counterstained with Harris' hematoxylin were then observed using a Nikon Eclipse microscope.

### Immuno-assays

Blood from live animals was collected by puncture of the maxillary artery with a Goldenrod lancet (Medipoint, USA). To perform intestinal lavages, the gut section extending from the stomach to the cecum was cut from euthanized mice and flushed with 10 ml cold PBS containing protease inhibitors (Complete® from Roche plus 10 mM PMSF from Sigma). After centrifugation (10 min at 10.000 rpm), supernatants were collected and all samples were frozen until use. Bronchoalveolar lavages were performed by injection of PBS containing protease inhibitors in the cannulated trachea. To quantify IgG and IgA specific for *Yersinia* antigens by ELISA, microtiter plates (NUNC) were coated with 5 µg/ml of a sonicate of *Y. pseudotuberculosis* IP32953 (hereafter referred to as *Y. ptb* Ag.) grown at 37°C on LB agar, as described before [17]. To quantify F1–specific IgG and IgA, plates were coated with F1 antigen (10 µg/ml), as described previously [25]. After blocking plates with 5% dry milk in PBS containing 0.1% Tween 20, bacteria grown at 37°C were serially diluted in PBS containing 0.1% BSA and were incubated in wells. Bound antibodies were detected using horseradish peroxidase (HRPO)–coupled rat antibodies specific for mouse IgG (Becton-Dickinson Pharmingen) or IgA (Caltag Laboratories), and HRPO activity was revealed using TMB substrate (OptiEIA, BD Pharmingen). Antibody titers were calculated as the reciprocal of the lowest sample dilution giving a signal equal to two times the background.

### Cell-mediated response in vaccinated animals

Spleens taken aseptically from euthanized animals were cut in pieces and dissociated using cell strainers (BD Biosciences). Erythrocytes were lyzed using Gey's hemolytic solution [27] and splenocytes were extensively washed with cold PBS. Cells resuspended in RPMI 1640+Glutamax™ (Invitrogen) supplemented with 5% fetal bovine serum, penicillin/streptomycin and 10 mM ß-mercaptoethanol were laid in 24 wells plates (5×10^6^/well) and stimulated with either *Y. ptb* Ag. (5 µg/ml), the F1 antigen (5 µg/ml) or Concanavalin A (1 µg/ml; Sigma) as a positive control. After three days, the supernatant was collected and the cytokine content was determined using IFNγ and IL-17 assays (Duosets, R&D Systems).

### Evaluation of mouse protection against a challenge with *Y. pestis*

The fully virulent *Y. pestis* strain CO92 or its non-encapsulated derivative CO92Δ*caf1* (Table 1) were grown at 28°C and suspensions in saline containing 10^5^ or 10^7^ cfu (33×LD_50_ or 3,300×LD_50_, respectively) were prepared. Mice vaccinated or not 28 days before were anesthetized and were infected by instillation of 10 µl of *Y. pestis* suspensions in the nostrils. Animal survival was monitored for 21 days.

### Statistical analyses

The Log-rank (Mantel-Cox) test was used to compare survival curves (protection). The non-parametric Mann-Whitney test was used to compare lesions, weight, antibody titers and cytokines production.

## Results

### Construction of an attenuated variant of *Y. pseudotuberculosis* IP32953

Strain IP32953 was chosen to generate an irreversibly attenuated *Y. pseudotuberculosis* strain because its genome has been determined [20]. The HPI, encoding the Yersiniabactin iron capture system [28], was deleted by allelic exchange, generating IP32953ΔHPI (Table 1). The LD_50_ of IP32953ΔHPI (LD_50_ oral = 6.8×10^8^) was 16 times higher than that of the parental IP32953 (4.2×10^7^ cfu). Since more attenuation was required for a vaccine, the chromosomal *psaA* gene encoding the pH 6 Ag pilus [29]) and the virulence plasmid-borne *yopK* gene, encoding the type three secretion system effector protein YopK [30], were additionally deleted. The triple deletant (ΔHPI, ΔPsaA, ΔYopK) generated, named V674, exhibited a strong virulence attenuation (LD_50_>3×10^9^ cfu), which was comparable to that of strain IP32680 [17]. Mice receiving 10^8^ cfu of V674 presented no signs of disease and no weight loss (Fig. S1A), whereas the parental IP32953 induces severe signs of disease and weight loss before death [17]. Vaccination with a single oral dose (10^8^ cfu) of V674 conferred protection to 69% of mice against an intranasal challenge with the fully virulent *Y. pestis* strain CO92 at a dose lethal for naive animals (10^5^ cfu = 33×LD_50_). Although this level of protection was superior to that of IP32680 (30%; [17] it was considered insufficient, and V674 was further modified.

### Construction of the V674pF1 strain producing an F1 capsule

**Figure 1:**
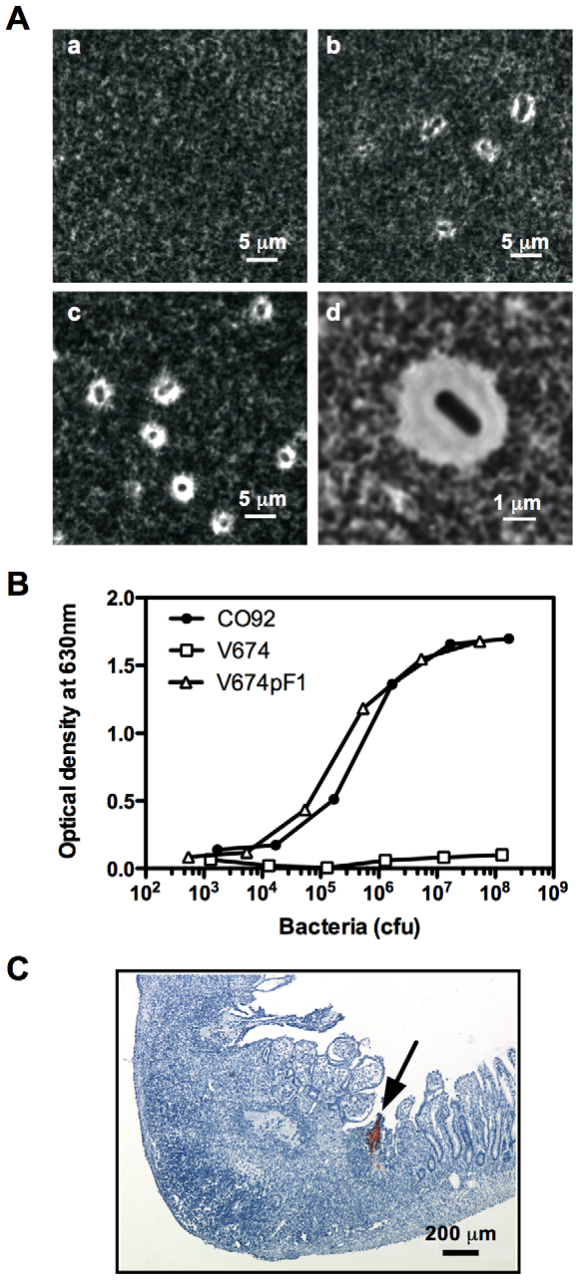
Strain V674pF1 produces an F1 capsule. A: Strains (a) V674 as negative control, (b) CO92 as positive control, and (c–d) V674pF1 in India ink observed under phase-contrast microscopy. The capsule excludes the India ink and appears as a clear halo around bacterial cells. B: Comparison of F1 production by V674, V674pF1 and CO92 by ELISA. C: F1 production by V674pF1 *in vivo*: Peyer's patches were taken from animals infected orally 5 days before with 10^10^ cfu of V674pF1, and subjected to an immunohistological staining with an anti-F1 mouse mAb, followed by a hematoxylin counterstain. An example of a bacterial focus (brown-red color) is indicated by an arrow.

In order to increase the ability of the candidate vaccine strain to induce a protective immunity against *Y. pestis*, V674 was further engineered to produce the *Y. pestis* F1 capsule. The *caf* operon from CO92, required for surface production of the F1 antigen in *Y. pestis*, was cloned into the pGEN plasmid. The resulting pGEN-*caf* plasmid was introduced into V674, generating V674pF1 (Table 1). The formation of a capsule around V674pF1 bacterial cells was observed (Fig. 1A c&d). When measured using an F1-specific ELISA, F1 production by V674pF1 was identical to that of *Y. pestis* CO92 (Fig. 1B). To determine whether the F1 capsule was produced *in vivo*, immunohistological staining of F1 was performed on sections of Peyer's patches taken from mice having received V674pF1 five days before. Small foci of F1-positive bacteria were detected using an anti-F1 monoclonal antibody (Fig. 1C), while no staining was detected when an irrelevant and isotype-matched biotinylated mAb was used as control (data not shown). The V674pF1 was thus able to produce the F1 capsule within mouse tissues. To determine whether the production of F1 had an impact on the virulence of the vaccine strain, graded doses of bacteria were inoculated orally to mice. Mice having received V674pF1 (10^8^ or 10^9^ cfu) presented no signs of disease and no weight loss (Fig. S1B) and no lethality was observed. A high dose of 2×10^10^ cfu also caused no lethality, revealing a very strong attenuation of virulence.

### *In vivo* persistence and infectivity of recombinant strains

**Figure 2:**
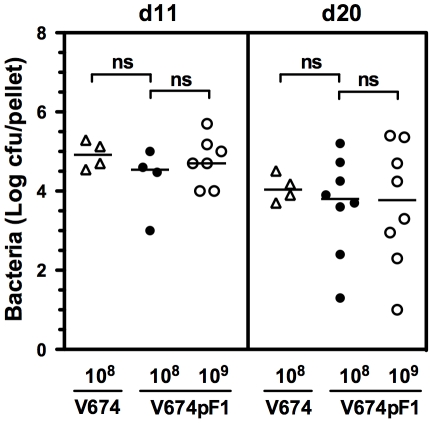
Gut colonization by *Y. pseudotuberculosis* mutants. Bacteria present in the feces (2 pellets) from mice orally vaccinated with strains V674pF1 and V674 at the indicated doses (cfu) were counted eleven and twenty days after. Shown are individual values from 4 to 8 mice per condition. ns: not significant.

The ability of V674pF1 to persist in the intestinal tract after oral inoculation was examined by counting bacteria present in feces (Fig. 2). At the vaccine dose of 10^8^ cfu, amounts of V674pF1 found at day 11 (d11) were comparable to those previously noted for the virulent IP32953 (#10^5^ cfu; [17], and were also comparable to those found with the non-encapsulated V674, indicating that attenuation of virulence or F1 production did not affect the ability of V674pF1 to colonize the gut. Levels of V674pF1 were comparable when a higher vaccine dose (10^9^ cfu) was used. The bacteria could be detected in feces for at least 20 days post infection, although at decreasing levels as compared to d11.

**Figure 3:**
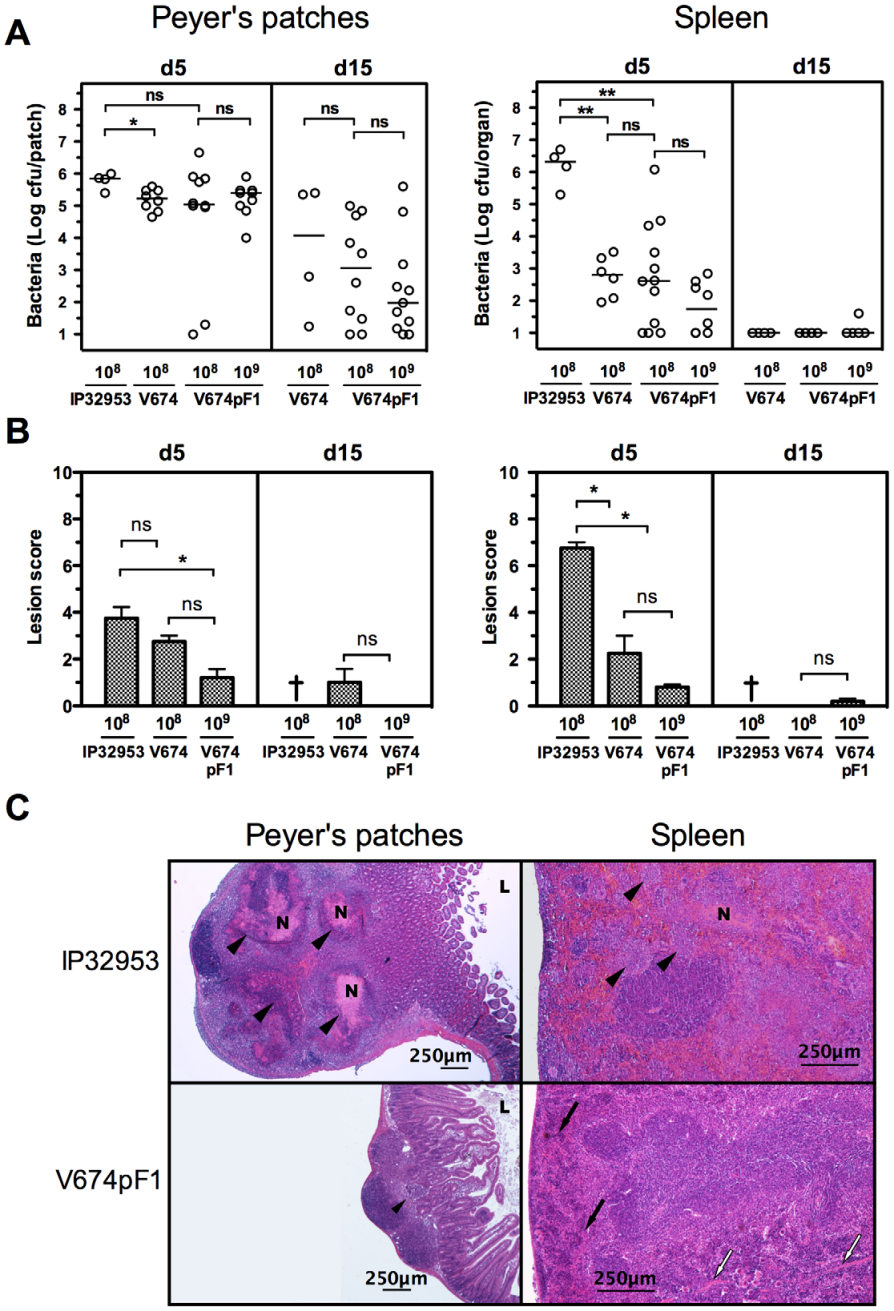
Dissemination and tissue lesions caused by *Y. pseudotuberculosis* mutants. A: bacteria present in the Peyer's patches and the spleen (cfu/organ) of mice infected orally with strains IP32953 (10^8^ cfu), V674 (10^8^ cfu) or V674pF1 (10^8^ and 10^9^ cfu). Shown are results from individual mice. The median is indicated by a horizontal line. B: quantification of lesions in Peyer's patches and spleen of mice infected orally with strains IP32953 (10^8^ cfu), V674 (10^8^ cfu) or V674pF1 (10^9^ cfu). The limit of detection was 10 cfu per organ. Organs taken at the indicated time were analyzed by histology after hematoxylin-eosin staining. Tissue lesion scores were recorded and shown are means ± s.e.m of four mice per condition. *: p≤0.05, ns: not significant, †: dead mice. C: Examples of lesions observed at Day 5 in the spleen and Peyer's patches from mice inoculated orally with IP32953 or V674pF1 (10^8^ cfu). Arrowheads indicate abscesses, and an N indicate an area of necrosis. In IP32953-infected spleen, hemorrhage is widespread, whereas in V674pF1 infected spleen only subcapsular microhemorrhages (black arrows) and congestioned blood vessels (white arrows) are observed. L: insestinal lumen.

To evaluate the dissemination of V674pF1 toward internal organs, its presence in Peyer's patches and spleen was examined (Fig. 3A). In Peyer's patches, amounts of V674pF1, or V674 were similar on D5, indicating that the production of F1 did not modify its ability to infect this lymphoid tissue. Similar loads were observed with a higher V674pF1 dose (10^9^ cfu). Ten days later (D15), lower levels of V674pF1 were observed, indicating a progressive lessening of infection, in agreement with counts seen in feces. Because animals infected by IP32953 died before D15, they could not be compared. In a deep organ such as the spleen, amounts of attenuated V674pF1 or V674 found 5 days after inoculation were significantly low as compared to IP32953, in agreement with their attenuation of virulence (Fig. 3A). Again, neither the presence of F1 nor the dose of V674pF1 used affected the splenic load. Ten days later, V674pF1 or V674 were most often not detectable, showing that the attenuated bacteria were rapidly cleared (Fig. 3A).

The potential development of lesions induced by the bacteria in these target organs was examined. As a reference, the wild type IP32953 at the 10^8^ cfu lethal dose caused severe lesions to both the spleen and Peyer's patches (abscesses, necrosis), together with signs of erosion of the flanking intestinal mucosa (scores >6/10; Fig. 3 B&C). In contrast, the same dose of V674 strain caused mainly congestion and microhemorrhages in the spleen and liver, and infrequent abscesses (scores <2/10, Fig. 3 B) in all three tissues tested. When used at a high dose of 10^9^ cfu to maximally reveal potential harmful effects, theV674pF1 strain also caused mild tissue lesions (scores ≤1/10; Fig. 3 B&C) that were not significantly different from those caused by V674, in spite of the dose difference. In agreement with bacterial clearance, histology of the spleen and Peyer's patches of mice vaccinated with either attenuated strain was normal or almost normal on Day 15 (score <1; Fig. 3 B), showing that tissues had healed. Altogether, our observations demonstrate that V674pF1 inoculated orally is able to colonize the gut and to interact with Peyer's patches, but fails to disseminate to high levels in the spleen, confirming its very high attenuation.

### Humoral immune response elicited by vaccination

**Figure 4:**
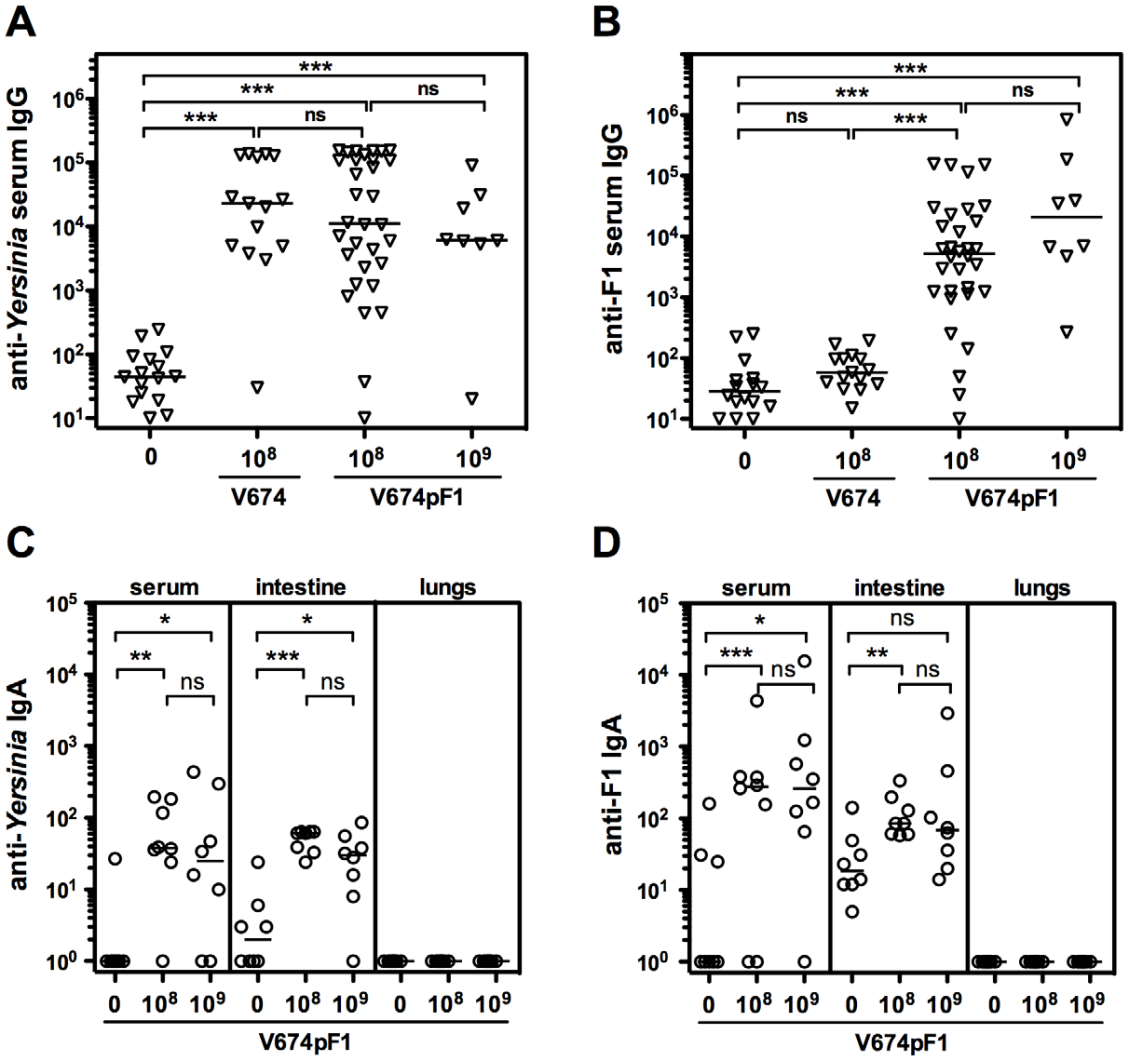
Seric and mucosal humoral immune response of vaccinated mice. To determine serum IgG titers against *Y. pseudotuberculosis* antigens (A) or against purified F1 antigen (B), blood was taken on day 21 from mice vaccinated orally with either V674 (10^8^ cfu; 16 mice) or V674pF1 (32 mice received 10^8^ cfu, 8 mice received 10^9^ cfu), or from naive animals (8 mice). To determine anti-*Y. pseudotuberculosis* (C) or anti-F1 (D) IgA levels in serum, intestine and lungs, groups of 8 mice received 10^8^ cfu or 10^9^ cfu of strain V674pF1 orally, or were not vaccinated, and were sacrificed 3 weeks later to collect blood, intestinal lavage (5 ml) and bronchoalveolar lavage (2 ml). Each dot represents an individual animal and medians (-) are shown. *: p<0.05, **: p<0.005, ***: p<0.001, ns: not significant.

High levels of serum IgG directed against *Y. pseudotuberculosis* antigens were detected in sera from mice having received one oral dose of V674pF1 (10^8^ cfu) 21 days before but not in sera from naive mice (Fig. 4A). Increasing the vaccine dose to 10^9^ cfu did not significantly increase IgG. Comparison with mice vaccinated with the non-encapsulated V674 strain revealed comparable anti-*Yersinia* IgG levels (Fig. 4A). High levels of IgG directed against F1 were detected in sera from mice vaccinated with V674pF1, and not in sera from unvaccinated or V674-vaccinated mice.

Because immunization through the oral route was expected to induce a mucosal type of immune response, IgA directed against both F1 and other *Yersinia* antigens were measured in mucosal tissues and blood after vaccination with V674pF1. Significant amounts of IgA were detectable in both intestinal lavages and sera but not in bronchoalveolar lavages (Fig. 4 C&D). Increasing the vaccine dose from 10^8^ cfu to 10^9^ cfu did not significantly affect the levels of IgA observed.

### Cellular immune response to *Yersinia* antigens elicited by vaccination

**Figure 5:**
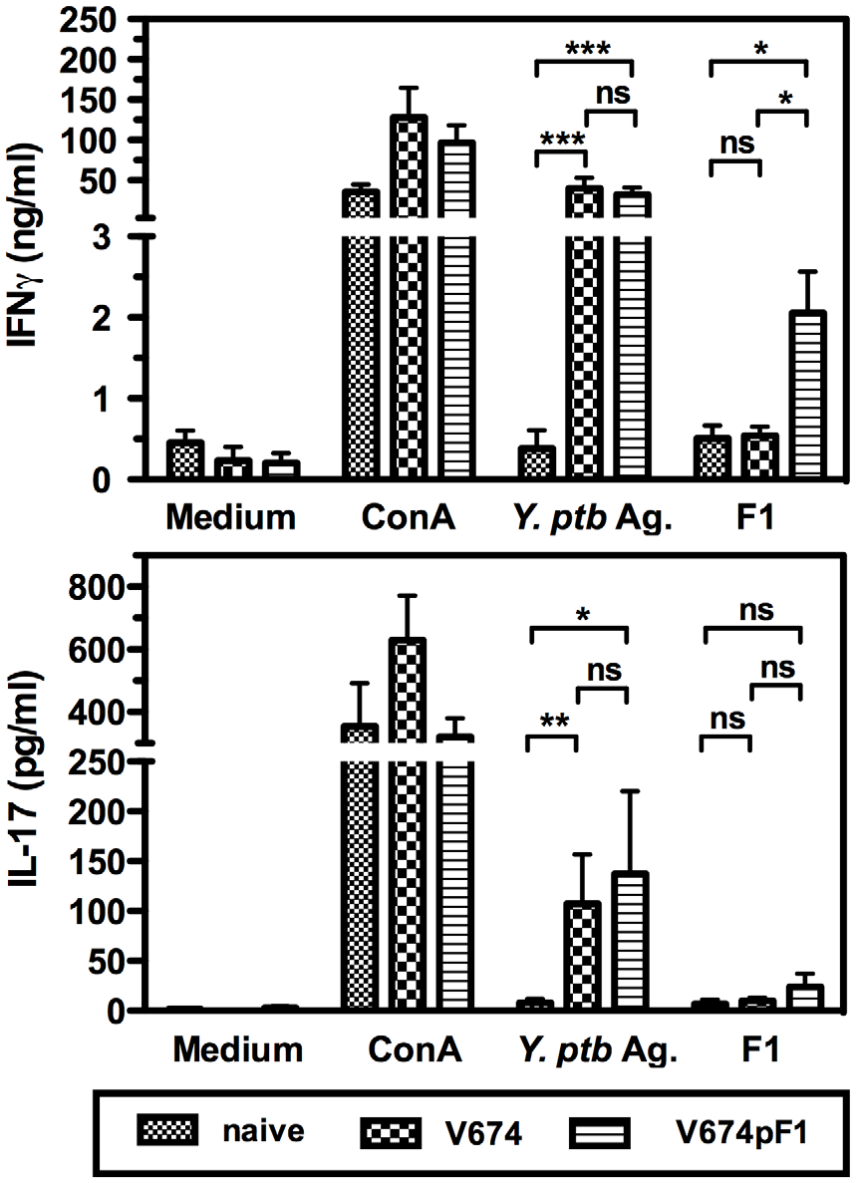
Cellular immune response of vaccinated mice. Splenocytes isolated from mice vaccinated orally 21 days before with strains V674pF1 (10^8^ cfu) or V674 (10^8^ cfu) or unvaccinated mice (naive) were stimulated *in vitro* with 5 µg/ml of either a *Y. pseudotuberculosis* antigenic preparation (*Y. ptb* Ag.) or purified F1 antigen. The mitogen Concanavalin A (ConA: 1 µg/ml) served as positive control. Supernatants taken 3 days after stimulation were tested by ELISA for the presence of IFNγ (A) and IL-17 (B). Shown are the mean ± s.e.m. of 16 mice per condition (2 pooled experiments). *: p<0.05, **: p<0.005, ***: p<0.001, ns: not significant.

The ability of V674pF1 to induce a cellular immune response was evaluated by comparing cytokine production by splenocytes taken from animals vaccinated with V674pF1 or V674 (both at 10^8^ cfu) 21 days before, or not vaccinated. Splenocytes were stimulated with either a *Y. pseudotuberculosis* antigenic preparation obtained by sonication, or purified F1 antigen. The mitogen ConA was used as a positive control (Fig. 5 A&B).

Cells from mice vaccinated with either V674pF1 or V674 significantly produced IFNγ in response to *Y. pseudotuberculosis* antigens, whereas cells from control naive mice did not (Fig. 5A), revealing an antigen-specific memory response. Comparable levels of IFNγ for the V674pF1 and V674 groups indicated that the presence of F1 in V674pF1 did not affect the development of the cellular response against other *Yersinia* antigens. Cells from V674pF1–vaccinated mice also produced IFNγ in response to F1, whereas cells from naive or V674-vaccinated mice did not, indicating a F1-specific cellular response.

IL-17 production was also examined because IL-17-producing T lymphocytes (Th17 cells) are key players of antibacterial mucosal immunity [31], [32], [33], [34]. Splenocytes from V674pF1- and V674-vaccinated mice produced IL-17 upon stimulation with *Y. pseudotuberculosis* antigens (Fig. 5B), whereas cells from naive mice did not, indicating the recruitment of *Y. pseudotuberculosis*–specific IL-17 producing cells. Because splenocytes from only half of the mice vaccinated with V674pF1 produced IL-17 upon stimulation by F1, the mean production was not statistically significant. Notably, *Y. pseudotuberculosis* antigens induced a stronger production of IFNγ and IL-17 than F1 (×15 and ×6 respectively), indicating that the multiple antigens included are important targets of cell-mediated immunity.

### Protection against pneumonic plague

**Figure 6:**
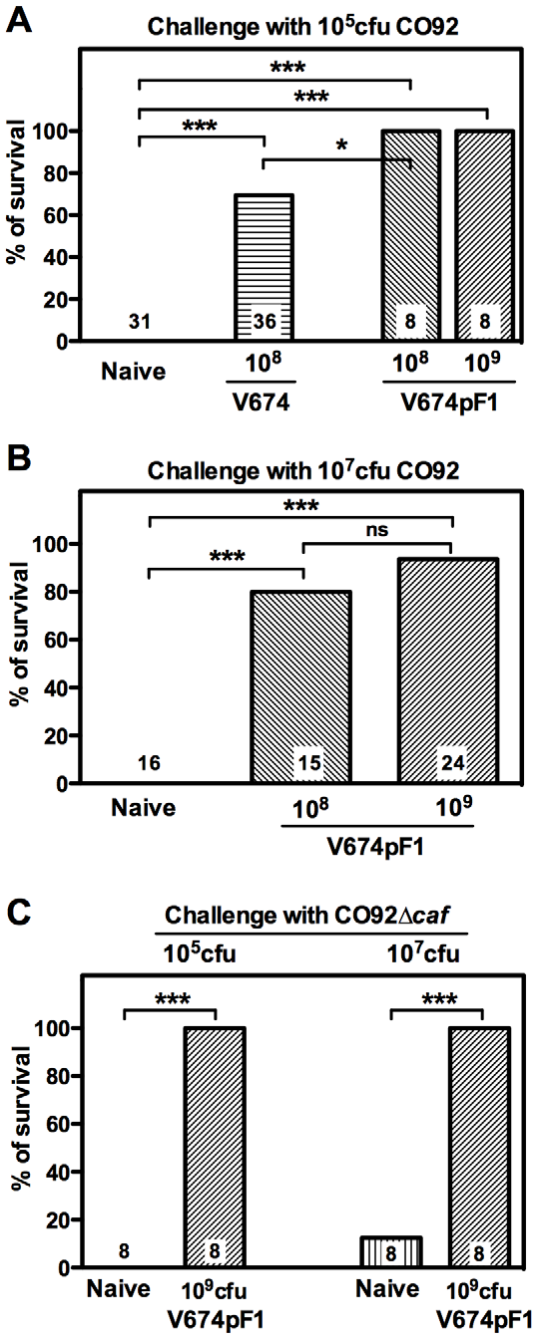
Protection of vaccinated mice against pneumonic plague. Mice having received a single oral vaccination with strains V674pF1 or V674 at the indicated doses were challenged 4 weeks later by intranasal instillation of the indicated dose of *Y. pestis* CO92 (A, B) or CO92Δ*caf* (C). Mouse survival was recorded daily for 21 days. Results from repeated experiments with 7–8 animals per group were pooled and the total number of mice per condition is indicated inside the corresponding bar. *: p≤0.05, **: p≤0.005, ***: p<0.001. ns: not significant.

The ability of V674pF1 to confer protective immunity was evaluated by challenging immunized mice intranasally with a lethal dose of the fully virulent *Y. pestis* CO92 (10^5^ cfu = 33×LD_50_). While all unvaccinated animals died within 3–4 days (Fig. 6A), a single oral inoculation of V674pF1 (10^8^ cfu) resulted in complete (100%) protection. In contrast, vaccination with V674 conferred protection to 69% of animals only. When a very high challenge dose of 10^7^ cfu CO92 (3,300×LD_50_) was used to mimic a severe contamination, mice vaccinated with 10^8^ cfu of V674pF1 showed 80% protection and this protection reached 94% when a vaccine dose of 10^9^ cfu was administered (Fig. 6B).

Finally, we evaluated whether the immunity induced by V674pF1 was protective against a virulent F1-negative *Y. pestis*. To this aim, a CO92Δ*caf1 Y. pestis* was constructed. This mutant had an LD_50_ comparable to that of CO92 by the intranasal route (LD_50_ = 5.6×10^3^ cfu as compared to 2.8×10^3^ cfu for CO92). Mice vaccinated orally with 10^9^ cfu of V674pF1 completely resisted a challenge infection (100% survival) by CO92Δ*caf* at both the 10^5^ cfu infectious dose and the high 10^7^ cfu dose (Fig. 6C).

## Discussion

Our initial study demonstrated that the naturally attenuated *Y. pseudotuberculosis* strain IP32680 could serve as an oral vaccine against bubonic plague [17]. However, this strain had weaknesses that precluded its development for human use. Because its genome had not been analyzed, its naturally low virulence could have resulted from a point mutation, with the possibility that a reverse mutation could restore virulence. In addition, IP32680 inoculated orally conferred only a weak level of protection (30%) against the pneumonic form of plague [17], after two vaccine doses. The goals of the present work were therefore twofold: first, to obtain a strain whose attenuation was irreversible and well characterized, and second to provide high-level protection against pneumonic plague. The first objective was reached by using strain IP32953, whose genome has been sequenced [35], and to attenuate it by deleting three major virulence genes, thus preventing reversion to virulence.

The genetically attenuated *Y. pseudotuberculosis* V674 strain provided 69% protection against pneumonic plague, and therefore was clearly more efficient than our previous IP32680 strain. However, it was still less efficient than attenuated *Y. pestis* strains used as plague vaccines in the past such as EV76 [36] or recently proposed engineered strains [37], [38]. Since the pioneer studies of Meyer, the F1 antigen composing the *Y. pestis* capsule is recognized as a major target of protective immunity due to its abundance and easy access at the bacteria surface [39]. F1 is absent from *Y. pseudotuberculosis*, and to enhance the efficiency of V674, the second step of our work was to introduce the *caf* operon coding for F1 into V674. With the presence of F1 at its surface, V674pF1 conferred full protection (100%) against pneumonic plague. Even against a very high intranasal challenge dose of CO92 (10^7^ cfu, i.e. 3,300×LD_50_), V674pF1 had an excellent protective score (94%). V674pF1, after a single oral dose, thus reached a level of protection that had, to our knowledge, not been reported for other live vaccines, including attenuated *Y. pestis* strains [37], [40], [41] or recombinant vectors producing *Y. pestis* antigens such as *Salmonella* [11], [42] or viruses [12], [43].

The F1 capsule is not an essential virulence factor for *Y. pestis* in mammals (reviewed by [44]), as shown by the fact that F1-negative *Y. pestis* mutants are still mortal for mice, primates and humans ([45], [46], the present work) although it is required to achieve full pathogenicity in certain mouse strains [47]. *Y. pestis* virulence is recognized to be multifactorial, so that the transfer of a single gene in an avirulent *Y. pseudotuberculosis* was unlikely to increase its virulence. Indeed, high oral doses of V674pF1 induced no lethality, in agreement with similar observations in F1-producing *Salmonella* candidate vaccines [11].

The live attenuated vaccines have several advantages over subunit vaccines. Recently developed candidate vaccines against plague are composed of two antigens: the *Y. pestis*-specific capsular F1 antigen and the virulence plasmid-encoded V antigen (LcrV) common to the three pathogenic *Yersinia* species. Molecular and live vaccines based on F1 and V provide protection to mice against pneumonic plague [12], [13], [14], [16], however they confer only variable levels of protection to non-human primates [48]. Such molecular vaccines using Alum as adjuvant mainly induce antibody production against the F1 antigen [49], allowing virulent *Y. pestis* variants lacking the F1 antigen to escape from the protective immunity of anti-F1 antibodies [46]. In contrast, live vaccines are strong inducers not only of humoral immunity but also of cell-mediated immunity [48], an important component of protection against pneumonic plague [50]. We observed that splenocytes from mice orally vaccinated with both V674pF1 and V674 strongly produced IFNγ upon restimulation *in vitro* with *Y. pseudotuberculosis* antigens, indicating the development of a *Y. pseudotuberculosis*–specific cellular immunity. IFNγ typically characterizes the type 1 response critical for optimal vaccine-induced protection against *Y. pestis* infection. Indeed, it was previously shown that injection of IFNγ and TNFα protects mice against *Y. pestis* infection [51] and that neutralization of these cytokines abrogates vaccine-induced protection against pneumonic plague [52]. IFNγ activates phagocytes and help them destroy internalized bacteria. Therefore the potent IFNγ production by splenocytes from vaccinated mice observed in the present work may have such a role. The recruitment of *Yersinia*-specific cells producing IL-17 (Th17) was also observed. There is growing evidence that the development of Th17 cells is critical to vaccine-induced protection against mucosal infections by pathogenic bacteria, parasites, viruses and fungi [31], [53]. Indeed, IL-17 is a powerful inducer of PMN recruitment and release of antimicrobial peptides, and contributes to immunity induced against pneumonic plague by an attenuated *Y. pestis* candidate vaccine [34]. Because such effector Th17 cells can collaborate with Th1 lymphocytes [53], the induction of both subsets by V674pF1 may be a key of the high protection observed against *Y. pestis* in the lungs.

It is most notable that vaccination using the live attenuated V674pF1 *Y. pseudotuberculosis* strain provided full protection against pneumonic plague caused by a virulent *Y. pestis* strains lacking the F1 antigen, whereas the live attenuated *Y. pestis* KIM D27 (Δ*pgm*), used as vaccine in other studies, failed to protect [46], [49]. This inability of a live *Y. pestis* to protect was interpreted as resulting from a focalization of the immune response against the abundant F1 covering the bacteria, to the detriment of other antigens [49]. On the contrary, we show here that V674pF1 was able to trigger immunity simultaneously against F1 and the large array of target antigens common to *Y. pestis* and *Y. pseudotuberculosis*, as demonstrated by the comparable IgG titers against these antigens observed after vaccination with vaccine strains producing F1 or not. Moreover, the cellular response against *Yersinia* antigens was not only comparable after vaccination with V674 or V674pF1, but was also much stronger than that induced by the F1 antigen. This absence of focalization of the immune response on F1 thereby greatly enhances the likelihood of protection against a wide spectrum of *Y. pestis* variants. The contrast with studies using KIM D27 as vaccine [46], [49] cannot be ascribed to a difference in the amount of F1 capsule because V674pF1 produces as much F1 as *Y. pestis*. It could rather result from yet unidentified differences of immunogenicity between the two *Yersinia* species, or on the different routes of vaccination (intramuscular versus oral). In this regard, the F1 capsule also did not alter the capacity of the *Y. pseudotuberculosis* strain to settle in the intestinal tract. This was however not surprising because *Y. pestis* is virulent by the oral route, as shown by human plague cases after eating meat from an infected animal [54]. That V674pF1 was efficient through the oral route was an additional advantage because oral vaccination is both convenient, well accepted by persons, and avoids the risk of contamination through used syringes during mass vaccination.

In previous studies of mouse vaccination with F1-V subunit vaccines, repeated injections were required to obtain full protection [14], [16]. In contrast, full protection against pneumonic plague was obtained in the present work after vaccination with a single oral dose of V674pF1. The capacity of live vaccines to stimulate immunity for an extended period of time was the likely key to this efficiency. V674pF1 given by the oral route persisted for weeks in the gut, allowing a prolonged antigen presentation to the immune system.

F1 production significantly improved the performance of V674pF1 compared to V674, consistent with the development of F1-targeted effector mechanisms efficient in the lungs. The production of high amounts of IgG and IgA indicated that the humoral immune response was triggered at both systemic and mucosal levels. Antibodies contribute to defense against pneumonic plague, as shown by previous studies in which non-immune or immunodeficient mice were protected by instillation of anti-F1 antibodies in the airways [55], [56], [57]. Seric IgG have an easy access to the highly vascularized lung tissues and those induced by V674pF1 may have played such a role. Because immunization started at a mucosal surface, IgA could also have been actors of V674pF1-induced lung immunity [58]. No IgA could however be detected in bronchoalveolar lavages, showing that IgA were not necessary to protection.

In summary, this study demonstrated that a high level protection against pneumonic plague can be obtained by a single oral vaccination with the live attenuated *Y. pseudotuberculosis* V674pF1 producing the *Y. pestis* F1 capsule. Because the strain has been irreversibly attenuated by deletion of essential virulence factors, it colonizes the intestinal tract without causing lesions and stimulates both humoral and cell-mediated anti-plague immunity. Easy to administrate orally and costless to produce, this candidate vaccine is therefore well adapted to mass vaccination in endemic tropical regions, offering promising perspectives to control pneumonic plague mortality and transmission.

## Supporting Information

Figure S1 **Mice do not lose weight after oral inoculation of attenuated** ***Y. pseudotuberculosis*** **strains.** The weight of mice vaccinated orally at day 0 with strains V674 (A), or V674pF1 (B) at the indicated dose, or unvaccinated littermates (naive) was measured at regular intervals. Shown are means ± s.e.m. of 16 mice per group. No difference between groups at any given time was statistically significant.
(TIF)

Table S1 **Primers used in this study.**
(DOC)
