# Supplementary figures and images for: An Encapsulated Yersinia pseudotuberculosis Is a Highly Efficient Vaccine against Pneumonic Plague
Source: PLoS Negl Trop Dis. 2012 Feb 14;6(2):e1528. doi: 10.1371/journal.pntd.0001528 (PMC3279354; doi:10.1371/journal.pntd.0001528)

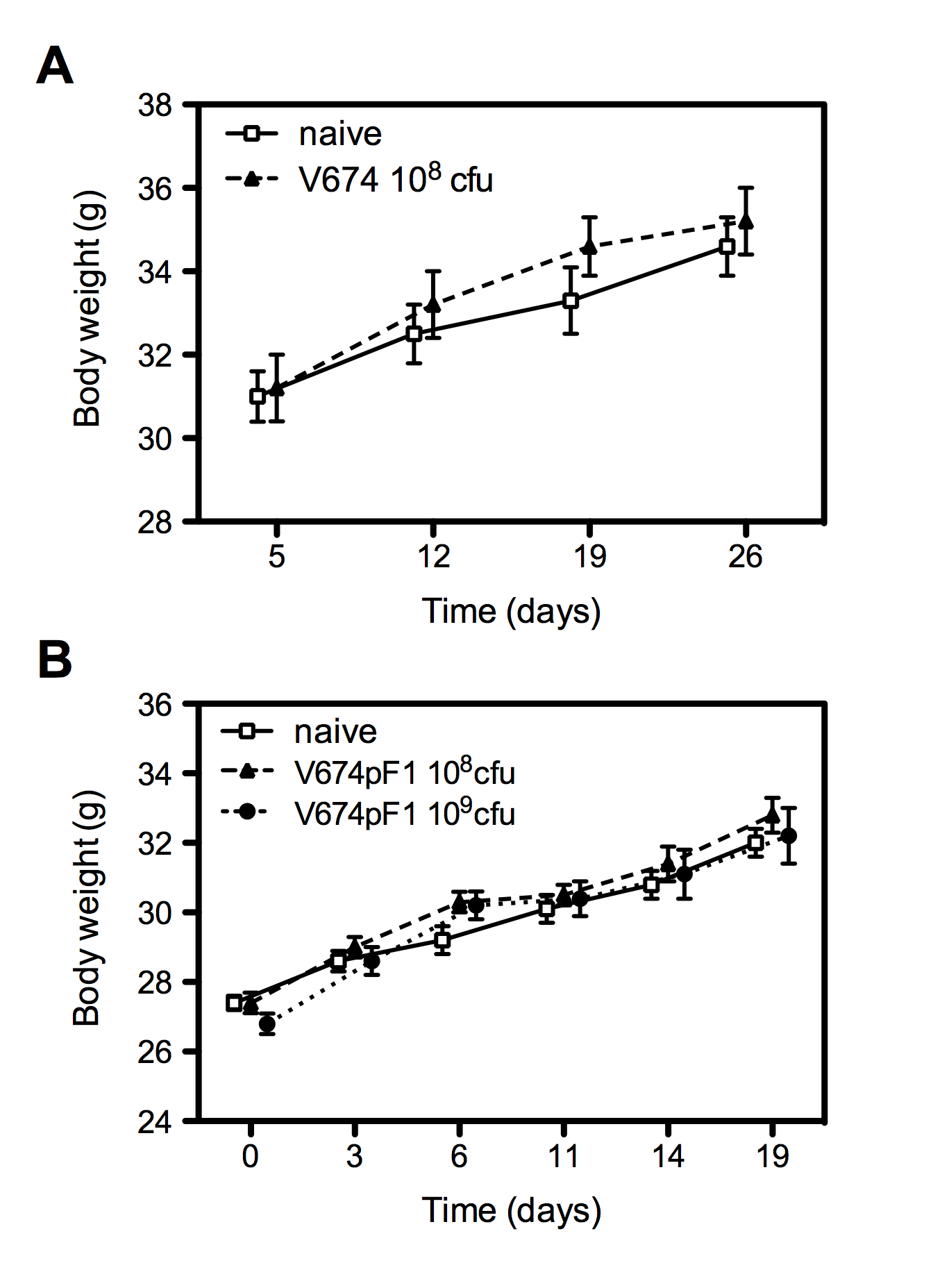

Supplement: Figure S1 — Mice do not lose weight after oral inoculation of attenuated Y. pseudotuberculosis strains. The weight of mice vaccinated orally at day 0 with strains V674 (A), or V674pF1 (B) at the indicated dose, or unvaccinated littermates (naive) was measured at regular intervals. Shown are means ± s.e.m. of 16 mice per group. No difference between groups at any given time was statistically significant. (TIF) [file pntd.0001528.s001.tif]
