# Supplementary material for: An Encapsulated Yersinia pseudotuberculosis Is a Highly Efficient Vaccine against Pneumonic Plague
Source: PLoS Negl Trop Dis. 2012 Feb 14;6(2):e1528. doi: 10.1371/journal.pntd.0001528 (PMC3279354; doi:10.1371/journal.pntd.0001528)
Supplement: Table S1 — Primers used in this study. (DOC) [file pntd.0001528.s002.doc]

Table S1. Primers used in this study

| **Target sequence** | **Primer number** | **Primer sequence** | **Purpose of the PCR** | **Source** |
| --- | --- | --- | --- | --- |
| **pUC4K** | 136A | 5’-GCAGGGGGGGGGGGGCGCT-3’ | To generate the Km cassette fragment for HPI mutagenesis in *Y. pseudotuberculosis* |  |
|  | 136B | 5’-CCTGCAGGGGGGGGGGGG-3’ |  |  |
|  | 166 | 5’-TCCTATGGAACTGCCTCGGT-3’ | To verify the Km cassette insertion after mutagenesis | This study |
|  | 167 | 5’-ATCGCGAGCCCATTTATACC-3’ |  |  |
| **pGP704-*km*** | 812 | 5’-ATTTTAAATAGATTAAGTACGTTAGGAATTATTACTTTCG  GCATGCTTAGTTTTGCAAAGCCACGTTGTGTCTCAAAATC-3’ | To generate the Km cassette flanked by short *caf* operon homologous regions for *Y. pestis caf1M*, *caf1A* and *caf1* deletion | This study |
|  | 814 | 5’-TGGTTAGATACGGTTACGGTTACAGCATCAGTGTATTTAC  CTGCTGCAAGTTTACCTCTGATTAGAAAAACTCATCG-3’ |  |  |
| **pGP704-*dfr*** | 460 | 5’-GGATTACTGACTTCATTGCT-3’ | To verify the insertion of the Tm cassette after mutagenesis | This study |
|  | 461 | 5’- CCTGTTGCGGCGCTTGAACGC-3’ |  |  |
|  | 544A | 5’- GCATATTCTACGCTATGTCGTAATAGCAATTATTACATTTAA  TGGAGACTGTTCTCTATCTGAGCTGTTGACAATTAATCATCC -3’ | To generate the trimethoprim cassette flanked by short *psaA* homologous regions for *Y. pestis psaA* mutagenesis | This study |
|  | 544B | 5’- AACAAAAACATACATAGAACATGACAGCAAGCCATCATGT  TCTATGATTACTCGTTTAGTTGATGCGTTCAAGCGCC -3’ |  |  |
| **pSW25** | 299B | 5’- TGGAACTCGCCGCCAGATTG-3’ | To verify the insertion of the Spec cassette after mutagenesis | This study |
|  | 354B | 5’-TGGCCGGATAACGCCAAGGTACG -3’ |  |  |
|  | 550A | 5’- ATGTTTATTAAAGATACTTATAACATGCGTGCTTTATGTA  CCGCTCTTGAACAGTCGCAATTGCGCCGAATAAATAC -3’ | To generate the spectinomycin cassette flanked by short *yopK* homologous regions for *Y. pestis* *yopK* mutagenesis | This study |
|  | 550B | 5’- CAGATAATTAACCTTTAACATAATCAACTACCATATCCCA  AACTCTTTAATATAGCTTCATTGGCTGGCACCAAGCAGTTTA -3’ |  |  |
| **HPI** | 420A | 5’- CCATTGCCCGTTTGGTTGTTGATAACAGCG -3’ | To generate the HPI upstream flanking region for the LFH procedure | This study |
|  | 420B | 5’- CGAGGCAGACCTCAGCGCCAAGAACCCTGCCTAGG -3’ |  |  |
|  | 421A | 5’- GATTTTGAGACACAACGTGGCTCCTCTGACTGGAC -3’ | To generate the HPI downstream flanking region for the LFH procedure | This study |
|  | 421B | 5’- CGGCCTTTATCTGTGTACCGATGCGCTACG-3’ |  |  |
|  | E | 5’- gacatgggtgaatataaccttcatagagcc -3’ | To verify HPI replacement by *km* when used with 166 | This study |
|  | F | 5’- cgataaagagatgagcatcatgctgtttc -3’ | To verify HPI replacement by *km* when used with 167 | This study |
|  | 194A | 5’- CCACGTTATGGCGCGGGAAGCAGCGTG -3’ | To verify the absence of the HPI (internal to *fyuA*) | This study |
|  | 137B | 5’- GCGCTGCTGCCGTTCATGTA -3’ |  |  |
| ***yopK*** | 549A | 5’- CTGCTCATTTACTGAGAACG -3’ | To verify the absence of *yopK* | This study |
|  | 549B | 5’- CTATAAGTAGAGAGTTTTTCGG -3’ |  |  |
|  | 414A | 5’- CACGAGTGCTGAGTACAAAATGTATCATGC-3’ | To verify *yopK* replacement by *dfr* when used with 354B | This study |
|  | 414B | 5’- CTGGGGAAGATGTTACGGTATACCCC-3’ | To verify *yopK* replacement by *dfr* when used with 354A | This study |
| ***Y. pestis* ∆*yopK* region** | 412A | 5’-GGTGTGCGCCAATCACCCCTTCGGTGAGGG -3’ | To generate the spectinomycin cassette flanked by long *yopK* homologous regions for *Y. pseudotuberculosis* *yopK* mutagenesis | This study |
|  | 413B | 5’- CCACAGCCGGAGAGGACTAATACTGAGGAC -3’ |  |  |
| ***Y. pestis* ∆*psaA* region** | 417A | 5’- GGGTACAAGGAGAACATATCCATACGTCC -3’ | To generate the spectinomycin cassette flanked by long *psaA* homologous regions for *Y. pseudotuberculosis* mutagenesis | This study |
|  | 418B | 5’- CGATCTTCAGGGAATTTTCCACCGGTC -3’ |  |  |
| ***psaA*** | 546A | 5’- CTGGTGAGGTGACTGTCAAGC -3’ | To verify the absence of *psaA* | This study |
|  | 546B | 5’- GCCTTAACGTACAGATGATC -3’ |  |  |
|  | 545A | 5’-GGCTTTATTGATGTCAATGG -3’ | To verify *psaA* replacement by *dfr* when used with 460 | This study |
|  | 545B | 5’-CAAGCATCACCGTTCTTAGG -3’ | To verify *psaA* replacement by *dfr* when used with 461 | This study |
| ***Y. pestis* ∆*psaA* region** | 417A | 5’- GGGTACAAGGAGAACATATCCATACGTCC -3’ | To generate the spectinomycin cassette flanked by long *psaA* homologous regions for *Y. pseudotuberculosis* *psaA* mutagenesis | This study |
|  | 418B | 5’- CGATCTTCAGGGAATTTTCCACCGGTC -3’ |  |  |
| ***caf* operon** | 837 | 5’- ATAAGAATGCGGCCGCGTGACTGATCAATATGTTGG -3’ | To clone the *caf* operon (from *caf1R* to *caf1*) | This study |
|  | 838 | 5’- CGTTAGGGCCCGTCAGTCTTGCTATCAATGC -3’ |  |  |
|  | 157A | 5’- CAGGAACCACTAGCACATC -3’ | To detect *caf1* |  |
|  | 157B | 5’-CCCCCACAAGGTTCTCAC -3’ |  |  |
|  | 771 | 5’-CGCACTAGTAATAGCATTCTAGATAGTGGGC-3’ | To verify *caf* replacement by *km* when used with 167 | This study |
|  | 815 | 5’-GATTAAGAAAAGCGAGGGTTAGGC-3’ | To verify *caf* replacement by *km* when used with 166 | This study |

References:

1. Pouillot F, Fayolle C, Carniel E. A putative DNA adenine methyltransferase is involved in *Yersinia pseudotuberculosis* pathogenicity. Microbiology 2007; 153:2426-34.

2. Tsukano H, Itoh K, Suzuki S, Watanabe H. Detection and identification of *Yersinia pestis* by polymerase chain reaction (PCR) using multiplex primers. Microbiol Immunol 1996; 40:773-5.
